# Homeostasis causes hallucinations in a hierarchical generative model of the visual cortex: the Charles Bonnet Syndrome

**DOI:** 10.1186/1471-2202-12-S1-P319

**Published:** 2011-07-18

**Authors:** David P Reichert, Peggy Series, Amos J Storkey

**Affiliations:** 1 School of Informatics, University of Edinburgh, Edinburgh, EH8 9AB, UK

## 

Hierarchical predictive models of the cortex [1,2] pose that the prediction of sensory input is a crucial aspect of cortical processing. Evaluating the internally generated predictions against actual input could be a powerful means of learning about causes in the world. During inference itself, rich high-level representations could then be utilized to resolve low-level ambiguities in sensory inputs via feed-back processing. A natural phenomenon to consider in such frameworks is that of hallucinations. In the Charles Bonnet Syndrome (CBS) [3-5], patients suffering from, primarily, eye diseases develop complex visual hallucinations containing vivid and life-like images of objects, animals, people etc. This syndrome is of particular interest as the complex content of the hallucinations rules out explanations based on simple low-level aspects of cortical organization, which are more suited to describe simpler hallucinations such as geometric patterns [6]. Moreover, the primary cause for the syndrome seems to be loss of sensory input in an otherwise healthy brain. Hence, a computational model of CBS needs to be capable of evoking rich internal representations under lack of external input, and elucidate on the underlying mechanisms.

We explore Deep Boltzmann Machines (DBMs) as models of cortical processing. DBMs are hierarchical, probabilistic neural networks that learn to generate the data they are trained on based on simple Hebbian learning rules. To explain CBS, we propose that homeostatic mechanisms that serve to stabilize neuronal firing rates [7] overcompensate for the loss of sensory input. With a model trained on simple toy images that then had its input removed, we demonstrate that homeostatic adaptation is sufficient to cause spontaneous occurrence of internal representations of the toy objects. We qualitatively analyze various properties of the model in the light of clinical evidence about CBS, such as an initial latent period before hallucination onset, an occasional localization of imagery to damaged regions of the visual field, and the effects of cortical suppression and lesions. To elucidate on the potential role of drowsiness in causing hallucinations, we model acetylcholine as mediating the balance between feed-forward and feed-back processing in the hierarchy.

An earlier version of this work was presented to a machine learning audience [8]. Here, we extend it with additional simulations to elaborate on our findings. In particular, we utilize more complex data sets, enforce sparsity to establish a clearer link between loss of input and decrease of cortical activity, and further justify the interpretation of the acetylcholine mechanism from a biological point of view.
